# Analysis of Surgical Mortality for Congenital Heart Defects Using RACHS-1 Risk Score in a Brazilian Single Center

**DOI:** 10.5935/1678-9741.20160022

**Published:** 2016

**Authors:** Candice Torres de Melo Bezerra Cavalcante, Nayana Maria Gomes de Souza, Valdester Cavalcante Pinto Júnior, Klébia Magalhães Pereira Castello Branco, Ronald Guedes Pompeu, Andreia Consuelo de Oliveira Teles, Rodrigo Cardoso Cavalcante, Giselle Viana de Andrade

**Affiliations:** 1 Hospital de Messejana Dr. Carlos Alberto Studart Gomes, Fortaleza, CE, Brazil.

**Keywords:** Congenital Heart Disease, Mortality, Risk Adjustment, Surgery

## Abstract

**INTRODUCTION:**

Risk Adjustment for Congenital Heart Surgery 1 (RACHS-1) score is a simple model that can be easily applied and has been widely used for mortality comparison among pediatric cardiovascular services. It is based on the categorization of several surgical palliative or corrective procedures, which have similar mortality in the treatment of congenital heart disease.

**OBJECTIVE:**

To analyze the in-hospital mortality in pediatric patients (<18 years) submitted to cardiac surgery for congenital heart disease based on RACHS-1 score, during a 12-year period.

**METHODS:**

A retrospective date analysis was performed from January 2003 to December 2014. The survey was divided in two periods of six years long each, to check for any improvement in the results. We evaluated the numbers of procedures performed, complexity of surgery and hospital mortality.

**RESULTS:**

Three thousand and two hundred and one surgeries were performed. Of these, 3071 were able to be classified according to the score RACHS-1. Among the patients, 51.7% were male and 47.5% were younger than one year of age. The most common RACHS-1 category was 3 (35.5%). The mortality was 1.8%, 5.5%, 14.9%, 32.5% and 68.6% for category 1, 2, 3, 4 and 6, respectively. There was a significant increase in the number of surgeries (48%) and a significant reduction in the mortality in the last period analysed (13.3% in period I and 10.4% in period II; *P*=0.014).

**CONCLUSION:**

RACHS-1 score was a useful score for mortality risk in our service, although we are aware that other factors have an impact on the total mortality.

**Table:** 

**Abbreviations, acronyms & symbols**	
CPB	= Cardiopulmonary bypass
RACHS-1	= Risk Adjustment for Congenital Heart Surgery 1
ROC	= Receiver operating characteristic
SPSS	= Statistical Package for Social Sciences

## INTRODUCTION

Congenital heart defects are the most common cause of congenital anomalies, representing a global health problem, with overall incidence of 28% of all major congenital anomalies^[1]^. The overall prevalence of congenital heart defects at birth increased substantially and is estimated at 9 per 1,000 live births over the last 15 years. This corresponds to 1.35 million newborns with congenital heart disease each year^[1]^.

The estimate for congenital heart disease in Brazil is 25,757 new cases per year. In 2010, 1,377 cases of births were registered with congenital heart defects in the DATASUS/Ministry of Health, representing 5.3% of the estimated to Brazil^[2]^.

Risk stratification in cardiac surgery is problematic due to large variations of the cases^[3,4]^. There are many surgical procedures that can be classified as palliative or corrective. Due to its high complexity, large number of congenital heart disease variants and few cases of each heart defect, it is difficult to establish a risk stratification system of nomenclature that is universally accepted^[3]^.

Special attention in the literature is given to the study by Jenkins et al.^[4]^, which propose an easily applicable risk score, called Risk Adjustment for Congenital Heart Surgery 1 (RACHS-1), which was based on the categorization of several surgical procedures, palliative or corrective, which had similar hospital mortality. Thus, the diseases have been divided into six categories according to the expected mortality for each^[4]^.

In order to review the mortality of surgical procedures performed by pediatric cardiology service of the Messejana Hospital during a period of 12 years of operation, its determinants and related factors, we used a comparison of the mortality of our institution with the expected mortality RACHS-1 risk score.

The aim of this study is to analyze the in-hospital mortality in children and adolescents after surgery for congenital heart defects from a pediatric cardiology service of a tertiary publish health center in Fortaleza, Ceará, Brazil, during its 12 years compared to RACHS-1 risk score.

## METHODS

After approval by the Research Ethics Committee and Institutional Research, we performed a retrospective analysis of data from records of cardiovascular surgeries performed from January 2003 to December 2014 at the Messejana Hospital, Fortaleza, Brazil. Our sample consisted of patients with congenital or acquired heart disease in the pediatric age group (under 18 years) who underwent cardiovascular surgery (corrective or palliative). Exclusion criteria were patients transferred to another service due to the impossibility of knowing the final outcome and patients who underwent procedures that could not be categorized for not belonging to the group of surgeries previously described by the score (130 patients). The independent variables were age, weight, gender, presence of trisomy 21, diagnosis, type of surgery, use of cardiopulmonary bypass (CPB), category of the RACHS-1 score. The dependent variable was the in-hospital mortality.

Based on the Statute of Children and Adolescents, the patients were divided into four categories according to age: neonatal (1-28 days), 1^st^ year of life (29 days to less than 1 year), children (1-12 years) and adolescents (12 years and to under 18 years of age). Hospital mortality was considered only for cases that occurred during hospitalization, regardless of time, for not being possible the full knowledge of the deaths occurred after discharge. The RACHS-1 risk score was used to classify the population according to surgical risk. Patients who underwent more than one procedure during the same hospitalization had the score considered as complex.

The research was divided into two periods to evaluate the evolution of the service in the last twelve years, due to the availability of data only from 2003. The period I is defined as the first six years, from 2003 to 2008 and the period II from 2009 to 2014. We evaluated the number of procedures performed, complexity of surgery and hospital mortality.

Data were analyzed using SPSS (Statistical Package for Social Sciences) statistical software, version 22 for Windows. Descriptive statistics were performed in relation to the distribution of variables and characteristics of the study population. Univariate analyzes were performed to assess the relationship between demographic and clinical variables and the outcome (mortality).

Continuous data were analyzed by statistical Klomogorov-Smirnov (KS) test for normality and the data considered normal were evaluated by Student's t-test and ANOVA; and categorical variables by the chi-square test (x^2^). Categorical variables were represented by absolute numbers and percentages (with a confidence interval to 95% of an estimate) and continuous data, by the mean or median with their respective dispersion measures. To assess the predictive capacity of RACHS-1 score for mortality, we used the Receiver Operating Characteristic (ROC) curve and the estimated area under it.

Because of the retrospective nature of the study, data on sex, age, mortality were not available for all patients. During the statistical analysis, some data crossing did not have similar number of total procedures.

## RESULTS

During the study period, 3201 surgeries were performed. Of these, 3071 were classified according to the RACHS-1 score and 130 procedures could not be categorized by the score. Of the patients, 1643 (51.7%) were male, aged less than 1 year of age 1523 (47.5%) and 2281 (71.1%) used CPB. The median weight was 8 kg (4 to 17.8 kg). Demographic characteristics are shown in Table 1.

**Table 1:** Demographic characteristics of patients undergoing cardiovascular surgery between 2003-2014.

**Variables**	**Rate, (%)**
**Gender**	
Female	1532 (48.3)
Male	1643 (51.7)
**Age**	
0-28 days	351 (10.9)
29 days to 1 year	1172 (36.6)
>1 year to 12 years	1331 (41.5)
>12 years to under 18 years of age	287 (9.9)
**Weight**	
Median (IQ)	8 kg (4-17.8 kg)
**Trisomy 21**	
No	3015 (94.5)
Yes	177 (5.5)
**CPB**	
With CPB	2281 (71.1)
Without CPB	917 (28.6)

IQ=inter-quartile range; CPB=cardiopulmonary bypass

**Fig. 1:**
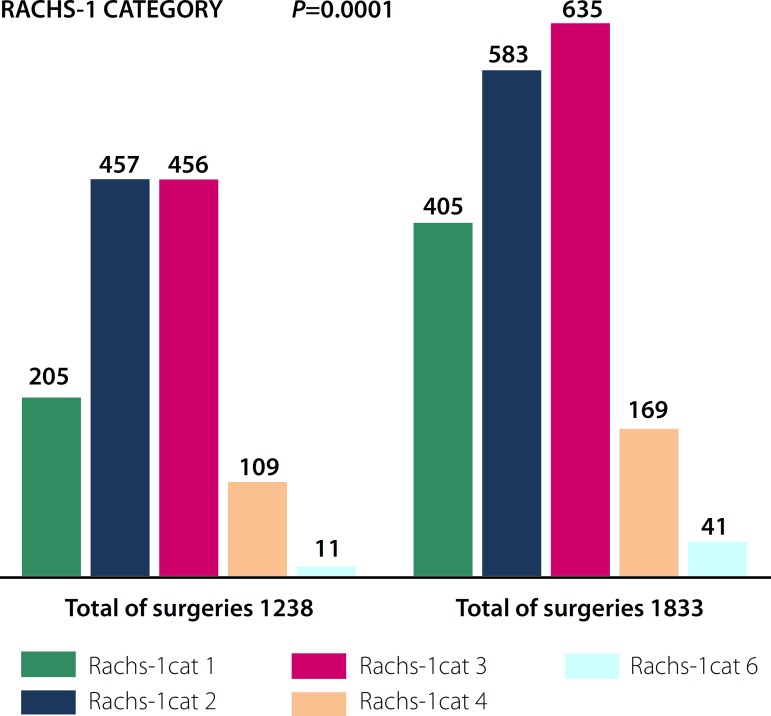
Distribution of surgeries according to the RACHS-1 score in both periods analyzed.

There was a significant increase in the number of surgeries in the last six years, from 1238 to 1833 procedures (*P*=0.001). This equates to a 48% increase in the total number of surgeries, with growth in all categories of RACHS-1 score (*P*=0.0001; Figure 1). Regarding the type of surgery, 2,281 (71.1%) used CPB and the most common category of RACHS-1 was the 3 (1091/3071, 35.5%).

### RACHS-1 Category - Total of Surgeries

**Fig. 2:**
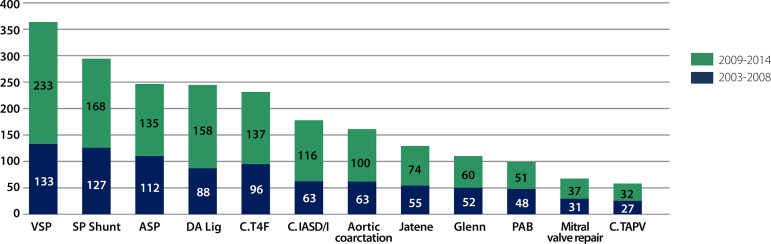
Distribution of the procedures according to the nomenclature proposed by RACHS-1 score in both periods. VSP=ventriculoseptoplasty; SP Shunt =systemic-pulmonary shunt; ASP=atrioseptoplasty; DA Lig.=ductus arteriosus ligation; C. T4F=tetralogy of Fallot correction; C. IASD/I=correction of full or intermediate atrioventricular septal defect; PAB=pulmonary artery banding; C. TAPV=correction of total anomalous pulmonary venous

The main procedures were ventriculoseptoplasty, pulmonary shunt, atrial septal defect and patent *ductus arteriosus* ligation (Figure 2).

Mortality also varied during the twelve years of records, with significant decrease despite an increase in the number of procedures, ranging from 13.3% (171/1288) to 10.4% (191/1889) in the period II (*P*=0.014). Mortality in the last three years is 9% (2012-2014).

When we evaluated the deaths according to RACHS-1 category, we found that the more complex the procedure, the higher the mortality rate is (*P*=0.0001), however when analyzing the association between RACHS-1 score and mortality in the two periods separately, we noted a decrease in mortality category in recent years, with the exception of category 6 (Table 2).

**Table 2:** Distribution of hospital mortality according to RACHS-1 score categories.

**RACHS-1 category**	**Rate, (%)**	**Deaths, n (%)**	Expected Mortality (*)
Category 1	610 (19.9)	11 (1.8)	0.4%
Category 2	1037 (33.9)	57 (5.5)	3.8%
Category 3	1088 (35.5)	164 (14.9)	8.5%
Category 4	277 (9.1)	88 (32.5)	19.4%
Category 5	_	_	_
Category 6	51 (1.7)	35 (68.6)	47.7%
Total	3071	352	_

**Fig. 3:**
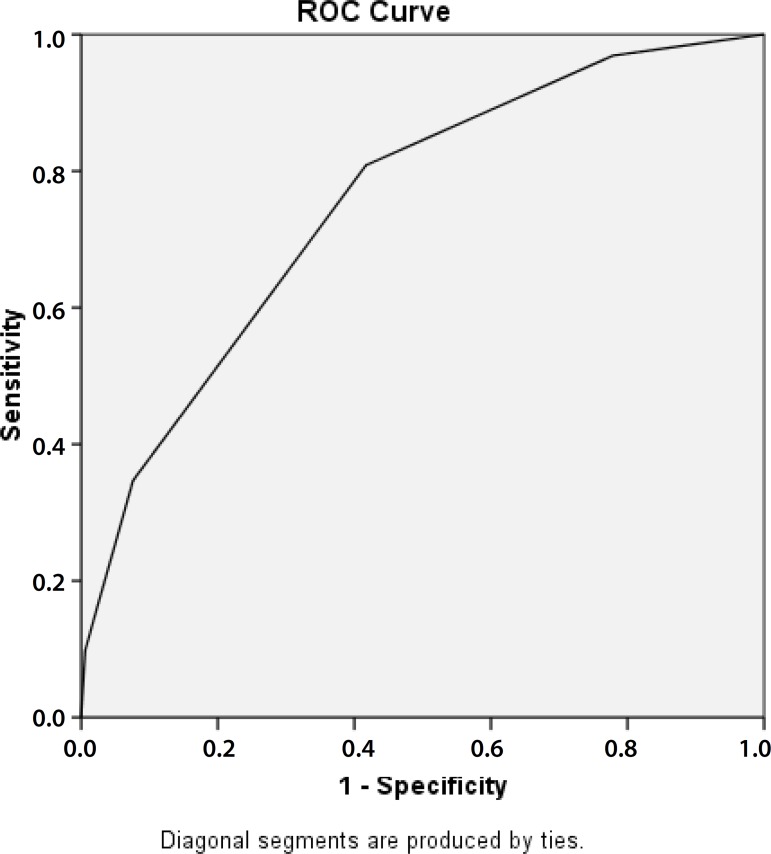
Discriminatory ability of RACHS-1 as a predictor of mortality.

In assessing the RACHS-1 score as a predictor of hospital mortality by ROC curve analysis, we find an area of 0.754 95% CI (0.727 to 0.78) (Figure 3).

After analyzing the deaths, the vast majority of patients belonged to the category of greater complexity (4 and 6), presenting lower weight and lower age (*P*=0.001; Table 3).

**Table 3:** Analysis of deaths from 2003 to 2014.

**Variables**	**Discharge, n (%)**	**Deaths, n (%)**	***P* value**
**RACHS-1**			
Categories 1, 2 and 3	2503 (91.5%)	232 (8.5%)	< 0.0001
Categories 4 and 6	205 (62.5%)	123 (37.5%)	
**Weight**			
Median (IQ)	9 kg (4.4 - 19 kg)	3.85 kg (3-7.7 kg)	< 0.0001
**Age**			
0-28 days	236 (76.8)	112 (32.2)	<0.0001
29 days to 1 year	1015 (86.8)	154 (13.2)	
1 year to 12 years	1243 (93.6)	83 (6.3)	
12 years to under 18 years of age	274 (95.5)	13 (4.5)	

IQ=inter-quartile range

We assessed the impact of each variable (RACHS-1 category, age, weight and the period in which the surgery was performed) on the outcome. Adjusting for the severity of the procedure, age, weight and the period in which the surgeries were performed showed direct relationship with the outcome. Children who had undergone surgery in the early years of service and with younger age and weight had higher mortality (OR 0.650, 95% CI 0.508 - 0.831; 4.983 95% CI 2.474 -10.038; OR 2.011 95% CI 1.546 - 2.616, respectively). The increase in each RACHS-1 category increases the risk of death by 135% approximately (Table 4).

**Table 4:** Predictors of mortality in multivariate analysis.

**Variable**	**OR (IC)**	***P* value**
For each increase of the RACHS-1 category	2.374 (2.074-2.717)	0.0001
Period from 2009 to 2014	0.650 (0.508-0.831)	0.0001
**Age**		
0 to 28 days	4.983 (2.474-10.038)	0.0001
29 days to 1 year	2.717 (1.388-5.317)	0.004
1 year to 12 years	2.091 (1.054-4.149)	0.035
12 years to under 18 years of age		
**Weight**		
0 to 5 kg	2.29 (1.673-3.151)	0.0001
5.1-10 kg	1.352 (0.928-1.970)	0.0001
>10 kg		

CI=confidence interval (95%)

## DISCUSSION

Congenital heart defects are the most common defects at birth, affecting about 1% of newborn^[1]^. National epidemiological studies have reported prevalence ranging from 5.94 to 9.58 per 1000 live births. However, few studies have been published with data on operative mortality or in-hospital patients with congenital heart diseases in Brazil^[5,6]^.

Due to the complexity and the high number of surgical procedures for correction of congenital heart diseases, comparisons between hospital or surgical mortality should respect a risk stratification^[7]^, without which, analysis on mortality would be inadequate.

The RACHS-1 score is a simple model that can be applied easily because it requires little data. Despite having some shortcomings as a low individual predictive power and disability of classification of all cardiac procedures^[8]^, it has been widely used to compare mortality among services and to evaluate the evolution of the quality of care provided^[9-11]^.

The research presents a series of the Messejana Hospital using RACHS-1 score for evaluation of the results of congenital heart disease treatments. The RACHS-1 score showed a good discriminatory ability for hospital mortality, with an area under ROC curve of 0.754, similar to previous studies in Brazil (Table 5). Cavalcanti et al.^[12]^ compared three severity scores (RACHS-1, Aristotle and STS-EACTS) of 360 patients operated in Pernambuco and concluded that the three stratification models showed similar discriminatory power for mortality. The total mortality of the sample was 14.7% with an area under ROC curve for RACHS-1 score of 0.738 (95% CI 0.690 to 0.783). In Sergipe, the RACHS-1 score was used to classify 932 procedures performed, with a predominance of category 1 and 2, with a mortality of 8.3% and area under ROC curve of 0.860 (95% CI 0.818 to 0.902) in 10 years of study follow-up^[13]^. Jacobs et al.^[9]^ performed a study review of 47 centers, including 45,635 procedures and found that the higher the complexity the higher the mortality rate was. Larsen et al.^[14]^ applied the RACHS-1 score to rank 957 procedures in Denmark and concluded that it is a good predictor of mortality and length of hospital stay.

**Table 5:** Summary of Brazilian activities with the use of RACHS-1.

**Reference**	**Local**	**Mortality**	**Duration of the study (years)**	**No. of patients**	**ROC curve**	**Mortality by RACHS-1 category**
Mattos et al.^[15]^, 2006	PE	14.7%	5	818	_	1 and 2 - 8.76% 3 and 4 - 26.12% 5 and 6 - 100%
Nina et al.^[16]^, 2007	MA	17.2%	3	145	_	1 - 3.8% 2 - 26% 3 - 60%
DATASUS- São Paulo, 2011^[17]^	SP	7.1%	1	974	_	1 - 1.3% 2 - 5.1% 3 - 10.4% 4 - 22.5% 5 - 60% 6 - 61.1%
Leite et al.^[13]^, 2012	PE	8.2%	9	932	0.860	1 - 0.25% 2 - 6.6% 3 - 11.1% 4 - 62% 6 - 100%
Cavalcanti et al.^[12]^, 2015	PE	14.7%	4.5	360	0.738	1 - 1.3% 2 - 11.4% 3 - 27.3% 4 - 50%
Current study, 2016	CE	11.6%	12	3071	0.754	1 - 1.8% 2 - 5.5% 3 - 14.9% 4 - 32.5% 6 - 68.6%

PE=Pernambuco; MA=Maranhão; SP=São Paulo; CE=Ceará

Although it is a good predictor of mortality, the RACHS-1 score does not address individual and structural factors of a service that can directly affect surgical outcomes. By comparing the data of deaths by category with the rates initially proposed by Jenkins et al.^[8]^, we found that the mortality of the service presents higher levels, but similar to other Brazilian studies (table 5). In a research performed in São Paulo in 2011, the observed mortality was 0.9%; 4.7%; 8.5%; 20.4%; 42.9% and 50%, in the categories 1, 2, 3, 4, 5 and 6, respectively^[17]^. It is necessary further analysis of the causes of deaths, but some comments can be cited in the evaluation of these numbers.

In developing countries, in addition to the complexity of the procedure, other determinants must be taken into account when assessing the mortality. About 90% of births worldwide occur in these countries and only a small portion of the population (7%) have access to treatment of congenital heart disease, by lack of resources in the area, leading to increases in morbidity and mortality^[18]^. This study was performed in a state in Northeastern Brazil, characterized by presenting various social, infrastructure and funding issues^[19,20]^. Due to late diagnosis and poor access to specialized tertiary center, patients are lately referred for surgery. These children usually have severe associated clinical conditions influencing the results as infection, malnutrition, severe cyanosis, ventricular dysfunction, renal dysfunction and pulmonary hypertension. These difficulties were also observed in other studies performed in the same region of the country (Cavalcanti et al.^[12]^, Mattos et al.^[15]^, Nina et al.^[16]^). Considering these aspects, Mattos et al.^[15]^ proposed the creation of a clinical-surgical score to predict intra-hospital mortality in this population. In addition to the complexity of the surgery, they assessed age, nutritional status, associated clinical factors (lung infection, heart failure, cyanosis, acidosis, infection, genetic syndrome, mechanical ventilation and prolonged hospitalization) and time of cardiopulmonary bypass. They concluded that the variables had direct influence on the outcome. In our analysis, we also found association between younger age and lower weight with mortality.

Even with advances in treatment occurred in the last four decades, the in-hospital mortality of our population is still high compared to rates reported in developed countries (Larsen et al.^[14]^; Oster et al.^[21]^; Nakayama et al.^[22]^). Despite the use of hospital mortality as a measure for evaluating cardiac surgery programs in adults and children, its use to compare services in pediatric patients is still problematic^[23]^. Welke et al.^[24]^, in a study performed in 2009, concluded that the mortality rate is not a suitable indicator for this purpose because the surgical results depend on many factors.

Our service can be characterized as high volume (250-349 procedures per year)^[24]^. The number of surgeries increased over the last three years to an average of 356.83 procedures/year. With this increase, the mortality rate showed a decrease to 10.5% and since 2012 is approximately 9%. These data are in agreement with the literature, which shows an inverse relationship between number of cases of pediatric cardiac surgery and mortality rates, becoming more evident with increasing complexity of the cases^[24]^. Centers with less than 150 cases per year have an increased risk of mortality (OR 1.59) and present worse results in difficult cases when compared to larger services^[25,26]^. However, Welke et al.^[24]^ demonstrated that this discriminatory capacity for mortality was adequate only after adjustment for individual associated risk factors and complexity of the cases, showing that the relationship between surgical volume and mortality is complex and multifactorial.

We observed a higher mortality in the first six years of our service, as well as the number of procedures, the early stage of establishment of a service also has direct influence on the results. There was a significant decrease in mortality in RACHS-1 categories from 1 to 4 in recent years. Mortality rates depend on the infrastructure, the learning curve for surgeons and experience of pediatric cardiologists and multidisciplinary team^[18]^. Nina et al.^[16]^ demonstrated the difficulties faced in the same region of the country in the early years of the service, with high mortality rates even for the simplest procedures. For better results, a highly qualified multi-disciplinary team and adequate institutional funding are required.

This publication had limitations because it is a single-center, retrospective study. Due to the nature of the research, our data were limited and some patients had no information on weight, age and outcome. We could not perform a more detailed analysis of the causes and risk factors associated with mortality. Further studies should be performed in order to understand and improve our results.

## CONCLUSION

The RACHS-1 score has a good ability to discriminate mortality, however, the analysis of mortality in developing countries requires the study of associated clinical factors, as well as structural and technological obstacles in our environment.

**Table:** 

**Authors' roles & responsibilities**	
CTMBC	Conception and design; operations and/or experiments performance; analysis and/or interpretation of data; statistical analysis; manuscript writing or critical review of its content; final approval of the manuscript
NMGS	Conception and design; interpretation of data; manuscript writing or critical review of its content
VCPJ	Manuscript writing or critical review of its content; final approval of the manuscript
KMPCB	Manuscript writing and/or critical review of its content; final approval of the manuscript
RGP	Manuscript writing and/or critical review of its content
ACOT	Final approval of the manuscript
RCC	Operations and/or; final approval of the manuscript
GVA	Operations and/or; final approval of the manuscript
